# Breastfeeding attitudes of Finnish parents during pregnancy

**DOI:** 10.1186/1471-2393-10-79

**Published:** 2010-12-02

**Authors:** Sari Laanterä, Tarja Pölkki, Anette Ekström, Anna-Maija Pietilä

**Affiliations:** 1 Piikivenkuja 5, 50600 Mikkeli, Finland; 2 Institute of Health Sciences, Faculty of Medicine, University of Oulu, PO Box 5000, FI-90014 University of Oulu, Finland; 3 School of Life Sciences, University of Skövde, PO Box 408, SE-54128 Skövde, Sweden; 4 Department of Nursing Science, Faculty of Health Sciences, University of Eastern Finland, Social and Health Care Center of Kuopio, PO Box 1627, FI-70211, Kuopio, Finland

## Abstract

**Background:**

Breastfeeding attitudes are known to influence infant feeding but little information exists on the prenatal breastfeeding attitudes of parents. The purpose of this study was to describe Finnish parents' prenatal breastfeeding attitudes and their relationships with demographic characteristics.

**Methods:**

The electronic Breastfeeding Knowledge, Attitude and Confidence scale was developed and 172 people (123 mothers, 49 fathers) completed the study. The data were analysed using factor analysis and nonparametric methods.

**Results:**

Breastfeeding was regarded as important, but 54% of the respondents wanted both parents to feed the newborn. The mean rank values of breastfeeding attitudes differed significantly when parity, gender, education, age, breastfeeding history and level of breastfeeding knowledge were considered. The respondents who were expecting their first child, were 18-26 years old or had vocational qualifications or moderate breastfeeding knowledge had more negative feelings and were more worried about breastfeeding than respondents who had at least one child, had a higher vocational diploma or academic degree or had high levels of breastfeeding knowledge. Respondents with high levels of breastfeeding knowledge did not appear concerned about equality in feeding.

**Conclusions:**

Both mothers and fathers found breastfeeding important. A father's eagerness to participate in their newborn's life should be included in prenatal breastfeeding counselling and ways in which to support breastfeeding discussed. Relevant information about breastfeeding should focus on the parents who are expecting their first child, those who are young, those with low levels of education or those who have gaps in breastfeeding knowledge, so that fears and negative views can be resolved.

## Background

The importance of breastfeeding on the health of both children and mothers is significant [1]. Breastfeeding rates (especially for 'exclusive breastfeeding') have not, however, reached their targets in several countries for example in the United Kingdom and the United States [2,3]. Thus, nursing studies have focused on the factors and interventions that affect the choice of feeding method, initiation of breastfeeding and duration [4-9]. One of the factors known to play a role is attitude towards infant feeding.

Mothers' breastfeeding attitudes are known to influence infant feeding choice [7,10]. According to parents, breastfeeding is recognised as being better for the newborn and a natural and cheap way to feed the infant [11,12]. Negative images or experiences about breastfeeding, including regarding breastfeeding as embarrassing, uncomfortable or painful [12-14], have been indicated as reasons for preferring bottle-feeding. Researchers have described the breastfeeding attitudes of health professionals [15-17], parents [18,19], students [20,21] and the general public [22]. Attitude is defined as a bipolar concept that has a cognitive, affective and behavioural component and is a response to a stimulus [23].

In this study, breastfeeding attitudes reflect people's views on infant feeding. Attitudes can appear stable, but can change. For example, a Swedish study indicated that the breastfeeding attitudes of health professionals could be shifted to show a more positive trend through educational intervention [17].

Previous studies have mostly focused on mothers' attitudes in the prenatal [12,24] or postpartum periods [14], but little information exists on both parents' prenatal breastfeeding attitudes [11]. However, since health professionals normally encounter pregnant parents, breastfeeding attitudes could be improved. A study in Taiwan indicated that breastfeeding attitude scores were higher after prenatal breastfeeding education intervention [25]. Furthermore, British studies have found that positive prenatal attitudes in mothers are linked to the intention and initiation of breastfeeding [24] and that attitude scores correlated with their spouses' scores [26]. Negative attitudes in fathers reduced breastfeeding initiation, according to a German study [27]. In addition, positive prenatal breastfeeding attitudes were linked to continued breastfeeding at least four weeks postpartum among teenage Canadian mothers [28]. An association between breastfeeding attitudes and duration was also found in an Australian study [29].

Studies conducted in the British Isles have indicated that the parents of breastfed infants have more positive attitudes towards breastfeeding than the parents of formula-fed infants [11]. Furthermore, breastfeeding mothers seem to be more supportive of breastfeeding than their spouses [30], but American studies have found that the breastfeeding attitudes of fathers can be more positive than the mothers predict [19,31]. The father's attitude is important because his role as a breastfeeding supporter is critical for the mother [32]. Intervention studies conducted in Italy and Brazil have indicated that the inclusion of fathers in breastfeeding promotion programmes has effects on the duration of exclusive breastfeeding and enhances maternal support [33,34]. Studying both parents' breastfeeding attitudes would, therefore, seem to be useful.

Studies on breastfeeding attitudes have been conducted in various cultures, but few studies [17,35] hail from Scandinavia. Breastfeeding initiation rates in Scandinavia are high [36]; for example, in Finland 99% of mothers initiate breastfeeding. Seventy-seven per cent of newborns, however, receive donor milk or artificial milk during their hospital stay [37]. Breastfeeding rates decrease rapidly following discharge and only 60% of one-month-old infants are exclusively breastfed [38], which in Finland means that the child receives only breast milk, vitamin D and possibly small amounts of water. At six months of age 60% of infants receive breast milk, but only 1% are exclusively breastfed [38], even though both the World Health Organization [39] and the Social and Health Ministry of Finland [40] recommend exclusive breastfeeding at this age. Consequently, information about parents' breastfeeding attitudes is necessary so that interventions to promote breastfeeding can be planned.

Breastfeeding attitudes are measured using different scales, mainly using paper forms. Breastfeeding in a public place or in front of others is a commonly tested issue [10,18,30,41]. One of the most frequently used instruments in attitude studies is the Iowa Infant Feeding Attitude Scale, which consists of items on the health and practical benefits as well as the financial benefits of breastfeeding [11,21,42-44]. However, the health benefits of breastfeeding derived from the attitude scale can overlap if breastfeeding knowledge and attitudes are measured simultaneously and with different scales. In addition, the father's role in breastfeeding has seldom been included in the attitude questions even though fathers are now actively involved in childcare [45]. Interviews [18], questionnaires using the Likert scale [26,30] and scenarios [30,41] have been used in studies on breastfeeding attitudes. The electronic data collection method, however, is rarely used to ascertain breastfeeding attitudes [46], even though young people are familiar with the Internet [47]. In addition, the relationship between breastfeeding attitudes and the intention [20,21] or initiation [14] of breastfeeding is commonly measured but differences in demographic characteristics have been less of a focus in breastfeeding attitude research [24]. Therefore, the aim of this study was to describe parents' prenatal breastfeeding attitudes using a web-based survey. The specific research questions were:

1. What kinds of attitudes do pregnant families have towards breastfeeding?; and

2. How do attitudes differ in relation to demographic characteristics?

## Methods

### Study design

This cross-sectional survey design was implemented to ascertain the breastfeeding attitudes of pregnant mothers and fathers. In Finland, all pregnant mothers are entitled to the free use of maternity healthcare clinics (MHCCs) [48]. This study was conducted in MHCCs and thereby as many mothers as possible were reached. The pregnant mothers visited the MHCCs about once a month and so the data collection period was five weeks. Thereafter, most of the mothers who visited the clinics received information about the study.

### Setting and participants

The study was conducted in south-east Finland because breastfeeding rates are low in this area [38]. Eight MHCCs were invited to participate in the study and the public health nurses (n = 19) at the clinics were asked to provide a covering letter describing the study to each family who visited the MHCC between 2 March and 3 April, 2009. The Internet address of the electronic form was provided in the written covering letter. The parents were asked to complete the electronic form separately, but if they did not have access to the Internet at home, or if they disliked the idea of completing an electronic form, they received paper forms that they could fill in at the MHCC and then return in a sealed envelope to the public health nurse. The public health nurses gave out 417 covering letters and 172 people completed the survey. Ten families out of the 417 (2%) did not have Internet access at home.

### The development of the scale

Attitudes were measured using the Breastfeeding Knowledge, Attitude and Confidence (BKAC) scale, which was developed on the basis of previous studies [18,22,30,35,41,49-53] for use in this study. Five breastfeeding experts evaluated the scale and no changes were made to the attitude items on the basis of their evaluations. The pretest was performed in February 2009. Minor changes such as alterations to the wording were made to the scale on the basis of the pretest (n = 8 pregnant mothers). The respondents reported that there were no ambiguous questions in the scale and it took approximately 15 minutes to complete the form.

The scale consisted of 26 knowledge, 25 attitude and 20 confidence items regarding breastfeeding. The knowledge items concerned practical issues, such as the initiation of lactation and complementary feeding. This article focuses on the attitude dimension, which was used to describe parents' basic attitudes to breastfeeding. In addition, there were 16 demographic questions to allow the dimensions to be considered in terms of background information. Three of the attitude items were scenarios and the rest of the 22 attitude items were measured on a four-point Likert scale (1 = strongly agree, 2 = somewhat agree, 3 = somewhat disagree, 4 = strongly disagree).

### Data analysis

The Statistical Package for Social Sciences (SPSS) statistical program, version 16.0, was used to analyse the results. The sum variables were produced on the basis of the factor analysis and their relationships with demographic characteristics were examined using the Kruskal-Wallis and Mann-Whitney U tests. If the Kruskal-Wallis test indicated a significant difference (*p *< .05), the Mann-Whitney U test with Bonferroni correction was used to discover which groups differed. A content validity index (CVI) and Cronbach's alpha coefficients were used to determine the reliability and validity of the scale.

### Ethical considerations

Ethical permission was obtained from the medical director of Mikkeli city and from the director of the public health service of Kouvola city who are responsible for decisions about ethical permission in the research area. The voluntary and anonymous nature of the study was indicated in the covering letter. The respondents received no financial reward from participation, but were entitled to a summary of the research findings if they wanted one [54,55].

## Results

### Description of the respondents

In all, 172 people (123 females and 49 males) returned the form. The mean age of all participants was 30.31 years (SD 5.79). Over half (53%) of the participants were expecting their first child. Twenty-nine per cent had one child and 18% had two or more children. Nearly all mothers (98%) had decided to breastfeed their baby and 4% lived without a spouse. The participants were asked about their breastfeeding histories, i.e. how many months they had been breastfed when they were babies. Altogether 72 mothers and 16 fathers knew the duration of breastfeeding. The average duration was 5.8 months (SD 4.5 months) for mothers and 7.4 months (SD 4.1 months) for fathers. This was not, however, a statistically significant difference. Breastfeeding knowledge was also tested using a 22-item questionnaire. The scores received ranged from four correct answers (18.2%) to 22 correct answers (100%). The means of the breastfeeding knowledge scores were 15.85 (SD 4.0) for mothers and 13.04 (SD 3.6) for fathers. The breastfeeding knowledge scores were classified into three categories: 5.8% had low (0-8 points) breastfeeding knowledge, 53.5% had moderate (9-16 points) breastfeeding knowledge and 40.7% had high (17-22 points) breastfeeding knowledge. The description of participants by gender is presented in Table 1.

**Table 1:** Description of the participants by gender

Demographics	Mothers	Fathers
	(n = 123)	(n = 49)
	%	%
Number of children		
0	48	65
1	33	18
2 or more	19	17
		
Highest educational level		
comprehensive school or matriculation	13.8	16
vocational qualification	40.7	39
higher vocational diploma or academic degree	44.7	45
missing	0.6	0
		
Personal income per month (net salary)		
≤ 1413 $ (1000 €)	26	20
1415-2827 $ (1001-2000 €)	50	32
≥ 2828 $ (2001 €)	23	45
missing	1	2
		
Smoking		
yes	8	22
no	92	76
missing	0	2
		
Had been breastfed oneself		
yes	88	71
no	5	4
don't know	7	25

### Pregnant parents' breastfeeding attitudes

A factor analysis using maximum likelihood and varimax rotation was performed [56]. An item was included if the factor loading was more than 0.30 and if the item had a greater than 0.30 correlation with at least one item. Altogether, four items with loading or a correlation of less than 0.30 were excluded. The maximum likelihood factor analysis with an unlimited number of factors produced five factors with >1.0 eigenvalues, and these explained 53% of the total variance. The factors and items are shown in Table 2. The first factor, 'Regarding breastfeeding as difficult', had loadings ranging from -0.888 to 0.870 and explained 16.6% of the variance. The second factor, 'Regarding breastfeeding as exhausting to the mother', explained 9.3% of the variance. The loadings ranged from -0.602 to 0.702. The third factor, 'Family-centred view on breastfeeding', had loadings ranging from 0.423 to 0.607 and explained 9.3% of the variance. The fourth factor was 'Equality in feeding' and explained 9.1% of the variance. This factor had loadings ranging from -0.687 to 0.830. The fifth factor, 'Worry about breastfeeding's negative impact on father', explained 8.7% of the variance with loadings ranging from 0.637 to 0.986.

**Table 2:** Parents' breastfeeding attitudes

Factors and items	Mean	(n = 172) Agree with the item		***α***^***f***^
		fr	*%*	
Factor 1 Regarding breastfeeding as difficult ^d^	3.0	33	*20*	*0.858*
breastfeeding is handy ^d ^*(loaded negatively to the factor)*	1.4	157	*93*	
breastfeeding is painful ^c^	3.1	31	*18*	
breastfeeding is easy ^c ^*(loaded negatively to the factor)*	2.1	128	*76*	
breastfeeding is difficult ^b^	3.0	43	*25*	
breastfeeding causes pressure ^a^	2.5	83	*49*	
Factor 2 Regarding breastfeeding as exhausting for the mother ^b^	2.3	105	*62*	*0.602*
the mother's own time is important ^b^	1.9	131	*77*	
breastfeeding gives strength to the mother ^a ^*(loaded negatively to the factor)*	2.4	96	*56*	
breastfeeding exhausts the mother ^a^	2.5	95	*56*	
Factor 3 Family-centred view on breastfeeding ^e^	1.3	165	*99*	*0.643*
important that the baby receives breast milk ^b^	1.3	162	*95*	
important that the family spends time together ^b^	1.1	169	*99*	
important that the spouse supports mother in breastfeeding ^b^	1.3	161	*95*	
breastfeeding brings joy to the mother ^b^	1.5	163	*96*	
breastfeeding brings joy to the baby ^b^	1.2	166	*98*	
Factor 4 Equality in feeding ^c^	2.3	101	*60*	*0.655*
both parents decide about the feeding method ^a^	2.1	118	*69*	
only the mother decides about the feeding method ^a ^*(loaded negatively to the factor)*	2.4	94	*55*	
important that both parents can feed the newborn ^a^	2.3	92	*54*	
Factor 5 Worry about breastfeeding's negative impact on father ^b^	3.3	19	*11*	*0.826*
worried about the father's relationship with the baby ^b^	3.4	18	*11*	
worried that the father feels himself to be an outsider ^b^	3.3	27	*16*	

^a ^one missing answer

^b ^two missing answers

^c ^three missing answers

^d ^four missing answers

^e ^five missing answers

^f ^Cronbach's alpha value

Over 95% of the respondents regarded it as important for their baby to receive breast milk, for the family to spend time together and for the spouse to support the mother in breastfeeding; they also thought that breastfeeding brings joy to the mother and to the baby. Forty-nine per cent, however, thought that breastfeeding puts pressure on the mother and 54% thought it important that both of the parents could feed the newborn baby. The means and the numbers of the agreed answers for the attitude items are shown in Table 2. Breastfeeding in front of others was presented with three scenarios and the division of the answers is shown in Table 3. Every third respondent (n = 172) regarded it inappropriate to breastfeed a one-year-old child in a hamburger restaurant.

**Table 3:** The division of the responses (n = 172) in the breastfeeding attitude scenarios

	Mothers (n = 123)			Fathers (n = 49)				Total (n = 172)		
Scenario	nes %	no %	I don't know %	Yes %	no %	I don't know %	p	yes %	no %	I don't know %
The father would like the mother to breastfeed because he thinks the breast milk would be the best for the baby. The mother does not want to breastfeed because she thinks it is too binding. Should the mother breastfeed the baby?	56	19	25	82	10	8	.006	63.4	16.3	20.3
Laura is breastfeeding when her friends (a man and a woman) come to visit. Should Laura move to another room to breastfeed?	15	78	7	18	76	6	ns.*	16	77	7
Kati is in the hamburger restaurant with her one-year-old baby. There are many customers and her meal is unfinished when the baby starts to cry. The baby is tired. Does Kati do right when she starts to breastfeed her baby at the table?	50	30	20	43	39	18	ns.	48	33	19

*Exact test was used

### The relationship between demographic characteristics and breastfeeding attitudes

A significant difference was evident in breastfeeding attitudes when gender, parity, age, education, knowledge and breastfeeding history were examined. No difference existed when income, smoking and area of residence were examined as characteristics. The mean rank values of the compared groups are presented in Table 4 (see Additional file 1, Group comparisons of breastfeeding attitudes). Table 5 indicates the responses to single items by gender and parity, which allowed specific information to be inferred (see Additional file 2, Breastfeeding attitudes by parity and gender).

## Discussion

The main finding was that parents regard breastfeeding positively but found it important that fathers can also participate in the infant feeding. In this sense, equality in feeding was a new aspect in breastfeeding attitude studies. It was evident that fathers wanted to be involved in choosing the feeding method and found it important to play an active role in feeding. The respondents who were expecting their first child regarded a joint parental decision about the newborn's feeding method as especially significant. Both parents valued breastfeeding and nearly all mothers intended to breastfeed. Previous studies have also indicated that mothers [24,26] who intend to breastfeed have partners [18] with positive attitudes towards breastfeeding.

There is scarce information about public breastfeeding in the Finnish culture. The majority of respondents regarded breastfeeding at home or in a public place as appropriate, but 33% did not regard the public breastfeeding of a one-year old child at a hamburger restaurant as acceptable. Studies conducted in other cultures have indicated that breastfeeding in front of others might be seen as embarrassing [57,58]. In the USA, public breastfeeding is protected by law e.g. in Arkansas [59]. Studies on African and Indian cultures have indicated that non-breastfeeding might be seen as evidence that the mother has been unfaithful or that she is HIV-positive [60,61]. Overall, public breastfeeding seems to be a culture-related issue.

Attitudes differed when parity was considered. The parents expecting their first child were more worried about the effect of breastfeeding on the father's relationship with the baby and his feeling of being an outsider than those who had at least one child. A Swedish qualitative study indicated that some first-time fathers experienced negative feelings when the mother was breastfeeding [45]. It is possible that the trend for equal parenthood means that breastfeeding is seen as a problematic issue. However, we noted that the parents who had at least one child did not think breastfeeding was as complicated as the parents who were expecting their first child did. This could imply that previous breastfeeding experience had been positive or that the respondents relied on their ability to resolve possible problems, whereas the parents who were expecting their first child had no prior experience on which to base their opinion.

Breastfeeding attitudes differed when education was considered. Sittlington and colleagues found the same result [24]. In the current study, respondents with a moderate level of education had more negative views on breastfeeding than those with a high level of education. No significant differences were found, however, when low levels of education were examined. This might be explained by the fact that some of those with the lowest level of education were students, e.g. at university, and they had not completed their education at the time of response.

According to this study, respondents over 27 years old regarded breastfeeding as less troublesome than those who were younger. Nonetheless, there were more parents who were expecting their first child in the youngest age group than in the other groups (*p *< .001) and they had no experience of breastfeeding. Therefore, some of them might have seen breastfeeding as a complicated method of feeding, although the reasons for this are unclear.

In addition, respondents with high levels of breastfeeding knowledge considered breastfeeding as less difficult and less exhausting than those with moderate or low levels of knowledge. This suggests that those who knew a lot about breastfeeding also had a very positive view of it.

The reliability of the BKAC scale is considered fairly good for the first measure because the Cronbach's alpha coefficients of the attitude dimension varied between 0.602 and 0.858 for each factor. The elimination of the item about the feeding of the newborn by both parents would have increased the Cronbach's alpha coefficient, but the item was regarded as relevant on the basis of experts' evaluations. Nevertheless, a Cronbach's alpha coefficient of over 0.6 has been described as acceptable [62]. The Cronbach's alpha coefficient was 0.84 in the knowledge dimension and 0.932 in the confidence dimension. The validity of the BKAC scale was measured using the CVI. Evaluations by all five breastfeeding experts rated 18 of the 21 attitude items as highly or quite relevant and thereby the scale-level content validity index, universal agreement calculation method was 0.857. The scale-level content validity index, averaging calculation method and item-level content validity index of the attitude items was 0.96, indicating the high validity of the dimension [63].

Nonetheless, this study has some limitations. First, the low response rate limits the generalisation of the results. Those who had poor computer skills or who were not interested in the research issue might have bypassed the study. Therefore, the results might indicate a misleadingly positive view about breastfeeding, although the high breastfeeding initiation rates in Finland would indicate that most mothers do regard breastfeeding as important. Furthermore, the small proportion of fathers needs to be taken into consideration. In this study, there were both fathers and mothers who regarded breastfeeding as not important and their responses seemed to be real; this implies that the respondents did not wilfully offer socially desirable answers. The response rate (21%) in this study was similar, however, to that of the web-based survey performed by Lucero and colleagues, in which 16.6% of paediatricians answered a questionnaire concerning attitudes, knowledge and clinical practices regarding breastfeeding and smoking [46].

A second limitation is that the families were informed only once about the study, so it is possible that some could have forgotten about it. The public health nurses said that some of the mothers placed the covering letter inside their maternity card and found it there the next time they visited the MHCC. In such cases, the public health nurses reminded the mothers and fathers to participate in the study if there was still time to respond. The participants were anonymous so the researchers had no opportunity to remind them. The chance to participate anonymously in the study might have increased interest in it, however.

The results of this study could be used in the development of breastfeeding counselling. The fathers' active attitudes towards and the image of breastfeeding of first-time parents need to be taken into account in clinical nursing. More studies are now required to examine equality in feeding. Follow-up studies are also needed to discover whether breastfeeding attitudes change between the prenatal and postnatal periods.

## Conclusions

The following conclusions were drawn on the basis of this study.

1. Pregnant Finnish parents have a positive attitude towards breastfeeding overall. The fathers' active attitudes towards feeding need to be considered in breastfeeding counselling and different ways to support the mother should be discussed.

2. Those expecting their first child, or were young, had vocational qualifications or moderate levels of breastfeeding knowledge had particularly negative feelings or were worried about breastfeeding. Therefore, breastfeeding counselling should focus on these groups and information about breastfeeding should be given prenatally.

3. The BKAC scale is a suitable instrument for the examination of parents' breastfeeding attitudes.

## Competing interests

The authors declare that they have no competing interests.

## Authors' contributions

SL participated in the design of the study, data collection, performed the statistical analysis and drafted the manuscript. AE contributed to the analysis and critically revised the manuscript. TP and A-MP participated in the design of the study, supervised the study and critically revised the manuscript. All authors have given their approval for the manuscript to be submitted in its present form.

## Pre-publication history

The pre-publication history for this paper can be accessed here:

http://www.biomedcentral.com/1471-2393/10/79/prepub

## Supplementary Material

Additional file 1 **Group comparisons of breastfeeding attitudes**. The mean rank values of compared groups Click here for file

Additional file 2 **Breastfeeding attitudes by parity and gender**. The responses to single attitude items by gender and parity Click here for file

## References

[B1] Stuebe AM Schwarz EB The risks and benefits of infant feeding practices for women and their children J Perinatol 2010 30 3 155 162 10.1038/jp.2009.107 19609306

[B2] Cattaneo A Burmaz T Arendt M Nilsson I Mikiel-Kostyra K Kondrate I Communal MJ Massart C Chapin E Fallon M Protection, promotion and support of breast-feeding in Europe: progress from 2002 to 2007 Public Health Nutr 2009 15 1 9 10.1017/S1368980009991844 19860992

[B3] Callen J Pinelli J Incidence and duration of breastfeeding for term infants in Canada, United States, Europe, and Australia: A literature review Birth: Issues in Perinatal Care 2004 31 4 285 292 10.1111/j.0730-7659.2004.00321.x 15566341

[B4] Bosnjak AP Grguric J Stanojevic M Sonicki Z Influence of sociodemographic and psychosocial characteristics on breastfeeding duration of mothers attending breastfeeding support groups J Perinat Med 2009 37 2 185 192 10.1515/JPM.2009.025 18956963

[B5] Thulier D Mercer J Variables associated with breastfeeding duration JOGNN: Journal of Obstetric, Gynecologic & Neonatal Nursing 2009 38 3 259 268 10.1111/j.1552-6909.2009.01021.x 19538614

[B6] Forster DA McLachlan HL Lumley J Factors associated with breastfeeding at six months postpartum in a group of Australian women Int Breastfeed J 2006 1 18 10.1186/1746-4358-1-18 17034645 PMC1635041

[B7] McMillan B Conner M Green J Dyson L Renfrew M Woolridge M Using an extended theory of planned behaviour to inform interventions aimed at increasing breastfeeding uptake in primiparas experiencing material deprivation Br J Health Psychol 2009 14 Pt 2 379 403 10.1348/135910708X336112 18980709

[B8] Amir LH Donath S A systematic review of maternal obesity and breastfeeding intention, initiation and duration BMC Pregnancy Childbirth 2007 7 9 10.1186/1471-2393-7-9 17608952 PMC1937008

[B9] Scott JA Binns CW Factors associated with the initiation and duration of breastfeeding: A review of the literature Australian Journal of Nutrition & Dietetics 1998 55 2 51 10197366

[B10] Kong SK Lee DT Factors influencing decision to breastfeed J Adv Nurs 2004 46 4 369 379 10.1111/j.1365-2648.2004.03003.x 15117348

[B11] Shaker I Scott JA Reid M Infant feeding attitudes of expectant parents: breastfeeding and formula feeding J Adv Nurs 2004 45 3 260 268 10.1046/j.1365-2648.2003.02887.x 14720243

[B12] Moore ER Coty M Prenatal and postpartum focus groups with primiparas: Breastfeeding attitudes, support, barriers, self-efficacy, and intention Journal of Pediatric Health Care 2006 20 1 35 46 10.1016/j.pedhc.2005.08.007 16399478

[B13] Flower KB Willoughby M Cadigan RJ Perrin EM Randolph G Family Life Project Investigative Team: Understanding breastfeeding initiation and continuation in rural communities: a combined qualitative/quantitative approach Matern Child Health J 2008 12 3 402 414 10.1007/s10995-007-0248-6 17636458 PMC2692345

[B14] Khoury AJ Moazzem SW Jarjoura CM Carothers C Hinton A Breast-feeding initiation in low-income women: Role of attitudes, support, and perceived control Womens Health Issues 2005 15 2 64 72 10.1016/j.whi.2004.09.003 15767196

[B15] Brodribb W Fallon A Jackson C Hegney D The relationship between personal breastfeeding experience and the breastfeeding attitudes, knowledge, confidence and effectiveness of Australian GP registrars Matern Child Nutr 2008 4 4 264 274 10.1111/j.1740-8709.2008.00141.x 18811791 PMC6860797

[B16] Clark A Anderson J Adams E Baker S Assessing the knowledge, attitudes, behaviors and training needs related to infant feeding, specifically breastfeeding, of child care providers Maternal & Child Health Journal 2008 12 1 128 135 10.1007/s10995-007-0221-4 17557200

[B17] Ekström A Widström A Nissen E Process-oriented training in breastfeeding alters attitudes to breastfeeding in health professionals Scand J Public Health 2005 33 6 424 431 16332607 10.1080/14034940510005923

[B18] Pollock CA Bustamante-Forest R Giarratano G Men of diverse cultures: knowledge and attitudes about breastfeeding J Obstet Gynecol Neonatal Nurs 2002 31 6 673 679 12465863 10.1177/0884217502239210

[B19] Freed GL Fraley JK Schanler RJ Attitudes of expectant fathers regarding breast-feeding Pediatrics 1992 90 2 Pt 1 224 227 1641286

[B20] Tarrant M Dodgson JE Knowledge, attitudes, exposure, and future intentions of Hong Kong university students toward infant feeding J Obstet Gynecol Neonatal Nurs 2007 36 3 243 254 10.1111/j.1552-6909.2007.00144.x 17489930

[B21] Marrone S Vogeltanz-Holm N Holm J Attitudes, knowledge, and intentions related to breastfeeding among university undergraduate women and men J Hum Lact 2008 24 2 186 192 10.1177/0890334408316072 18436970

[B22] Li R Rock VJ Grummer-Strawn L Changes in public attitudes toward breastfeeding in the United States, 1999-2003 Journal of the American Dietetic Association 2007 107 1 122 127 10.1016/j.jada.2006.10.002 17197280

[B23] Altmann TK Attitude: a concept analysis Nurs Forum 2008 43 3 144 150 10.1111/j.1744-6198.2008.00106.x 18715347

[B24] Sittlington J Stewart-Knox B Wright M Bradbury I Scott JA Infant-feeding attitudes of expectant mothers in Northern Ireland Health Educ Res 2007 22 4 561 570 10.1093/her/cyl113 17041021

[B25] Lin SS Chien LY Tai CJ Lee CF Effectiveness of a prenatal education programme on breastfeeding outcomes in Taiwan J Clin Nurs 2008 17 3 296 303 17931376 10.1111/j.1365-2702.2006.01927.x

[B26] Scott JA Shaker I Reid M Parental attitudes toward breastfeeding: their association with feeding outcome at hospital discharge Birth 2004 31 2 125 131 10.1111/j.0730-7659.2004.00290.x 15153132

[B27] Kohlhuber M Rebhan B Schwegler U Koletzko B Fromme H Breastfeeding rates and duration in Germany: a Bavarian cohort study Br J Nutr 2008 99 5 1127 1132 10.1017/S0007114508864835 18294424

[B28] Mossman M Heaman M Dennis CL Morris M The influence of adolescent mothers' breastfeeding confidence and attitudes on breastfeeding initiation and duration J Hum Lact 2008 24 3 268 277 10.1177/0890334408316075 18689714

[B29] Scott JA Binns CW Oddy WH Graham KI Predictors of breastfeeding duration: evidence from a cohort study Pediatrics 2006 117 4 e646 55 10.1542/peds.2005-1991 16585281

[B30] Shepherd CK Power KG Carter H Examining the correspondence of breastfeeding and bottle-feeding couples' infant feeding attitudes J Adv Nurs 2000 31 3 651 660 10.1046/j.1365-2648.2000.01320.x 10718885

[B31] Freed GL Fraley JK Schanler RJ Accuracy of expectant mothers' predictions of fathers' attitudes regarding breast-feeding The Journal of Family Practice 1993 37 2 148 152 8336095

[B32] Clifford J McIntyre E Who supports breastfeeding? Breastfeed Rev 2008 16 2 9 19 18767233

[B33] Pisacane A Continisio GI Aldinucci M D'Amora S Continisio P A controlled trial of the father's role in breastfeeding promotion Pediatrics 2005 116 4 e494 498 10.1542/peds.2005-0479 16199676

[B34] Susin LRO Giugliani ER Inclusion of fathers in an intervention to promote breastfeeding: impact on breastfeeding rates J Hum Lact 2008 24 4 386 392 10.1177/0890334408323545 18784322

[B35] Hannula L Imetysnäkemykset ja imetyksen toteutuminen. Suomalaisten synnyttäjien seurantatutkimus. [Perceptions of breastfeeding and the outcomes of breastfeeding - a follow-up study of Finnish mothers] PhD thesis 2003 University of Turku, Department of Nursing

[B36] Cattaneo A Yngve A Koletzko B Guzman LR Promotion of Breastfeeding in Europe project: Protection, promotion and support of breast-feeding in Europe: current situation Public Health Nutr 2005 8 1 39 46 10.1079/PHN2005660 15705244

[B37] Erkkola M Kronberg-Kippilä C Knip M Virtanen S Ravitsemus elämänkaaren alkupäässä: tavoitteisiin matkaa. [Hospital feeding, breastfeeding and weaning in the Finnish Diabetes Prediction and Prevention Nutrition Study] Suomen lääkärilehti - Finlands läkartidning 2006 61 48 5029 5035

[B38] Hasunen K Ryynänen S Imeväisikäisten ruokinta Suomessa vuonna 2005. [Infant feeding in Finland 2005] Ministry of Social Affairs and Health, Helsinki 2006 19 1 70

[B39] WHO The Optimal Duration of Exclusive Breastfeeding. Report of an Expert Consultation 2001 WHO, Switzerland

[B40] Hasunen K Kalavainen M Keinonen H Lagström H Lyytikäinen A Nurttila A Peltola T Talvia S Lapsi, perhe ja ruoka: imeväis-ja leikki-ikäisten lasten, odottavien ja imettävien äitien ravitsemussuositus. [The child, family and food. Nutrition recommendations for infants and young children as well as pregnant and breastfeeding mothers] Ministry of Social Affairs and Health, Helsinki 2004 11 1 254

[B41] Libbus MK Breastfeeding attitudes in a sample of Spanish-speaking Hispanic American women J Hum Lact 2000 16 3 216 220 10.1177/089033440001600306 11153155

[B42] De La Mora A Russell DW Dungy CI Losch M Dusdieker L The Iowa Infant Feeding Attitude Scale: Analysis of reliability and validity J Appl Soc Psychol 1999 29 11 2362 2380 10.1111/j.1559-1816.1999.tb00115.x

[B43] Scott J Shaker I Reid M Parental attitudes toward breastfeeding: Their association with feeding outcome at hospital discharge Birth 2004 31 2 125 131 10.1111/j.0730-7659.2004.00290.x 15153132

[B44] Binns CW Graham KI Scott JA Oddy WH Infants who drink cows milk: a cohort study J Paediatr Child Health 2007 43 9 607 610 10.1111/j.1440-1754.2007.01133.x 17688644

[B45] Fagerskiold A A change in life as experienced by first-time fathers Scand J Caring Sci 2008 22 1 64 71 10.1111/j.1471-6712.2007.00585.x 18269424

[B46] Lucero CA Moss DR Davies ED Colborn K Barnhart WC Bogen DL An examination of attitudes, knowledge, and clinical practices among Pennsylvania pediatricians regarding breastfeeding and smoking Breastfeed Med 2009 4 2 83 89 10.1089/bfm.2008.0119 19210131 PMC2981379

[B47] Changes in Internet Usage. Results from the 2008 Survey on ICT Usage http://www.tilastokeskus.fi/til/sutivi/2008/sutivi_2008_2009-04-27_tie_002_en.html

[B48] Laki sosiaali-ja terveydenhuollon asiakasmaksuista 1992 Finland 734 http://www.finlex.fi/fi/laki/ajantasa/1992/19920734

[B49] Williams EL Hammer LD Breastfeeding attitudes and knowledge of pediatricians-in-training Am J Prev Med 1995 11 1 26 33 7748583

[B50] Hull VJ Thapa S Wiknjosastro G Breast-feeding and health professionals: a study in hospitals in Indonesia Soc Sci Med 1989 28 4 355 364 10.1016/0277-9536(89)90037-3 2705008

[B51] Scavenius M van Hulsel L Meijer J Wendte H Gurgel R In practice, the theory is different: A processual analysis of breastfeeding in northeast Brazil Social Science & Medicine 2007 64 3 676 688 10.1016/j.socscimed.2006.09.002 17070973

[B52] Quintero Romero S Bernal R Barbiero C Passamonte R Cattaneo A A rapid ethnographic study of breastfeeding in the North and South of Italy Int Breastfeed J 2006 1 14 10.1186/1746-4358-1-14 16952324 PMC1570447

[B53] Dennis CL Faux S Development and psychometric testing of the Breastfeeding Self-Efficacy Scale Res Nurs Health 1999 22 5 399 409 10.1002/(SICI)1098-240X(199910)22:5<399::AID-NUR6>3.0.CO;2-4 10520192

[B54] World Medical Association Declaration of Helsinki http://www.wma.net/en/30publications/10policies/b3/index.html

[B55] Medical Reseach Act 1999 Finland 488 http://www.finlex.fi/en/laki/kaannokset/1999/en19990488.pdf

[B56] Burns N Grove SK The Practice of Nursing Research: Conduct, Critique, and Utilization 2005 Philadelphia: Elsevier/Saunders

[B57] Raisler J Against the odds: breastfeeding experiences of low income mothers J Midwifery Womens Health 2000 45 3 253 263 10.1016/S1526-9523(00)00019-2 10907335

[B58] Hannon PR Willis SK Bishop-Townsend V Martinez IM Scrimshaw SC African-American and Latina adolescent mothers' infant feeding decisions and breastfeeding practices: a qualitative study J Adolesc Health 2000 26 6 399 407 10.1016/S1054-139X(99)00076-2 10822181

[B59] Ogbuanu CA Probst J Laditka SB Liu J Baek J Glover S Reasons why women do not initiate breastfeeding: A southeastern state study Womens Health Issues 2009 19 4 268 278 10.1016/j.whi.2009.03.005 19589476 PMC2865685

[B60] Bassett MT Psychosocial and community perspectives on alternatives to breastfeeding Ann N Y Acad Sci 2000 918 128 135 10.1111/j.1749-6632.2000.tb05481.x 11131696

[B61] Rogers A Meundi A Amma A Rao A Shetty P Antony J Sebastian D Shetty P Shetty AK HIV-related knowledge, attitudes, perceived benefits, and risks of HIV testing among pregnant women in rural Southern India AIDS Patient Care STDS 2006 20 11 803 811 10.1089/apc.2006.20.803 17134354

[B62] Hair JF Anderson RE Tatham RL Black WC Multivariate Data Analysis 1998 Prentice Hall

[B63] Polit DF Beck CT The content validity index: are you sure you know what's being reported? Critique and recommendations Res Nurs Health 2006 29 5 489 497 10.1002/nur.20147 16977646

