# Supplementary material for: Breastfeeding attitudes of Finnish parents during pregnancy
Source: BMC Pregnancy Childbirth. 2010 Dec 2;10:79. doi: 10.1186/1471-2393-10-79 (PMC3003624; doi:10.1186/1471-2393-10-79)
Supplement: Additional file 1 — Group comparisons of breastfeeding attitudes. The mean rank values of compared groups [file 1471-2393-10-79-S1.DOC]

Table 4 Group comparisons of breastfeeding attitudes

| Characteristic | Sum scores | | | | |
| --- | --- | --- | --- | --- | --- |
|  | ‘regarding bf as difficult’  (mean rank value^1^) | ‘regarding bf as exhausting’  (mean rank value^1^) | ‘family-centred view on bf’  (mean rank value^1^) | ‘equality in feeding’  (mean rank value^1^) | ‘worry about bf’s negative impact on father’  (mean rank value^1^) |
|  |  |  |  |  |  |
|  |  |  |  |  |  |
| Gender^2^  women  men | *ns.* | *p*=.018  91.00  71.52 | *p*=.047  79.53  95.40 | *p*<.001  94.60  60.79 | *ns.* |
|  |  |  |  |  |  |
| Province^2^ | *ns.* | *ns.* | *ns.* | *ns.* | *ns.* |
|  |  |  |  |  |  |
| Parity^2^  parents who were expecting their first child  parents who had at least one child | *p*=.005  74.90  95.84 | *p*=.001  73.53  99.28 | *ns.* | *p*<.001  69.76  102.78 | *p*=.002  75.13  97.17 |
|  |  |  |  |  |  |
| Smoking^2^ | *ns.* | *ns.* | *ns.* | *ns.* | *ns.* |
|  |  |  |  |  |  |
| Breastfeeding history^2^  doesn’t know her/his history  does know her/his history | *ns.* | *ns.* | *p*=.002  114.25  79.88 | *ns.* | *ns.* |
|  |  |  |  |  |  |
| Income^3^ | *ns.* | *ns.* | *ns.* | *ns.* | *ns.* |
|  |  |  |  |  |  |
| Age^3^ | *p*=.017 | *p*=.029 | *ns.* | *ns.* | *ns.* |
|  |  |  |  |  |  |
| age^2,4^  ≤ 26 years  27–35 years | *p*=.018  55.93  75.36 | *p*=.021  57.16  76.08 | *ns.* | *ns.* | *ns.* |
|  |  |  |  |  |  |
| age^2,4^  ≤ 26 years  ≥ 36 years | *ns.* | *ns.* | *ns.* | *ns.* | *ns.* |
|  |  |  |  |  |  |
| age^2,4^  27–35 years  ≥ 36 years | *ns.* | *ns.* | *ns.* | *ns.* | *ns.* |
|  |  |  |  |  |  |
| Education^3^ | *ns.* | *p*=.035 | *p*=.033 | *p*=.005 | *p*=.026 |
|  |  |  |  |  |  |
| education^2,4^  low  moderate | *ns.* | *ns.* | *ns.* | *ns.* | *ns.* |
|  |  |  |  |  |  |
| education^2,4^  low  high | *ns.* | *ns.* | *ns.* | *ns.* | *ns.* |
|  |  |  |  |  |  |
| education^2,4^  moderate  high | *ns.* | *p*=.030  63.61  81.29 | *ns.* | *p*=.006  61.88  82.82 | *p*=.021  63.10  80.91 |
|  |  |  |  |  |  |
| Knowledge^3^ | *p*<.001 | *p*<.001 | *p*=.023 | *p*<.001 | *ns.* |
|  |  |  |  |  |  |
| knowledge^2,4^  low  moderate | *ns.* | *ns.* | *ns.* | *ns.* | *ns.* |
|  |  |  |  |  |  |
| knowledge^2,4^  low  high | *ns.* | *p*=.045  21.70  43.19 | *ns.* | *ns.* | *ns.* |
|  |  |  |  |  |  |
| knowledge^2,4^  moderate  high | *p*<.001  64.79  98.47 | *p*<.001  63.65  102.16 | *p*=.015  87.52  67.85 | *p*<.001  67.62  96.15 | *ns.* |

^1^ The lower mean rank values indicate stronger agreement to the sum score variable

^2^ Mann–Whitney U-test

^3^ Kruskal–Wallis test

^4^ Bonferroni correction
