# Supplementary material for: Breastfeeding attitudes of Finnish parents during pregnancy
Source: BMC Pregnancy Childbirth. 2010 Dec 2;10:79. doi: 10.1186/1471-2393-10-79 (PMC3003624; doi:10.1186/1471-2393-10-79)
Supplement: Additional file 2 — Breastfeeding attitudes by parity and gender. The responses to single attitude items by gender and parity [file 1471-2393-10-79-S2.DOC]

Table 5 Breastfeeding attitudes by parity and gender

| Items regarding breastfeeding attitudes  bf= breastfeeding | mothers  (n=123)  agree | | fathers  (n=49)  agree | | *p* |  | parents who were expecting their first child  (n=91)  agree | | parents who had at least one child (n=81)  agree | | *p* |
| --- | --- | --- | --- | --- | --- | --- | --- | --- | --- | --- | --- |
|  | fr | % | fr | % |  |  | fr | % | fr | % |  |
|  |  |  |  |  |  |  |  |  |  |  |  |
| Factor 1 Regarding bf as difficult |  |  |  |  |  |  |  |  |  |  |  |
| Bf seems to be handy. | 112 ^c^ | 93 | 45 ^a^ | 94 | *ns.* |  | 84 | 92 | 73 ^d^ | 95 | *ns.* |
| Bf seems to be painful. | 24 ^b^ | 20 | 35 ^a^ | 73 | *ns.* |  | 18 | 20 | 13 ^c^ | 17 | *p=.002* |
| Bf seems to be easy. | 94 ^b^ | 78 | 35 ^a^ | 73 | *ns.* |  | 57 | 74 | 61 ^c^ | 78 | *p=.002* |
| Bf seems to be difficult. | 34 ^a^ | 28 | 9 ^a^ | 19 | *ns.* |  | 23 | 25 | 20 ^b^ | 25 | *ns.* |
| Bf puts pressure on the mother. | 62 ^a^ | 51 | 21 | 43 | *ns.* |  | 50 | 55 | 33 ^a^ | 41 | *ns.* |
|  |  |  |  |  |  |  |  |  |  |  |  |
|  |  |  |  |  |  |  |  |  |  |  |  |
| Factor 2 Regarding bf as exhausting to the mother |  |  |  |  |  |  |  |  |  |  |  |
| I found it important that the mother has her own time after the baby is born. | 87 ^a^ | 71 | 44 ^a^ | 92 | *p=.031* |  | 80 | 88 | 51 ^b^ | 65 | *p<.001* |
| Bf gives strength to the mother. | 74 ^a^ | 61 | 22 | 45 | *ns.* |  | 47 | 58 | 49 ^a^ | 61 | *ns.* |
| Bf exhausts the mother. | 62 ^a^ | 51 | 33 | 67 | *ns.* |  | 58 | 64 | 37 ^a^ | 46 | *p=.015* |
|  |  |  |  |  |  |  |  |  |  |  |  |
|  |  |  |  |  |  |  |  |  |  |  |  |
| Factor 3 Family-centred view on bf |  |  |  |  |  |  |  |  |  |  |  |
| I find it important that my baby receives breast milk. | 117 ^a^ | 96 | 45 ^a^ | 94 | *ns.* |  | 87 | 96 | 75 ^b^ | 95 | *p=.004* |
| I find it important that the family spends time together when the baby is born. | 121 ^a^ | 99 | 48 ^a^ | 100 | *ns.* |  | 90 | 99 | 79 ^b^ | 100 | *p=.013* |
| I find it important that the spouse supports the mother in bf. | 116 ^a^ | 95 | 45 ^a^ | 94 | *ns.* |  | 85 | 93 | 76 ^b^ | 96 | *ns.* |
| Bf brings joy to the mother. | 115 ^b^ | 95 | 48 | 98 | *p=.032* |  | 90 | 99 | 73 ^b^ | 92 | *ns.* |
| Bf brings joy to the baby. | 119 ^a^ | 98 | 47 ^a^ | 98 | *ns.* |  | 89 ^a^ | 99 | 77 ^a^ | 96 | *ns.* |
|  |  |  |  |  |  |  |  |  |  |  |  |
|  |  |  |  |  |  |  |  |  |  |  |  |
| Factor 4 Equality in feeding |  |  |  |  |  |  |  |  |  |  |  |
| Choosing the feeding method for the newborn is a thing that the parents need to decide together. | 71 ^a^ | 58 | 47 | 96 | *p<.001* |  | 72 | 79 | 46 ^a^ | 58 | *p<.001* |
| Choosing the feeding method for the newborn is solely the mother’s decision. | 76 ^a^ | 62 | 18 | 37 | *p=.007* |  | 40 | 44 | 54 ^a^ | 68 | *p=.004* |
| I find it important that both parents can feed the newborn together. | 60 | 49 | 32 ^a^ | 67 | *ns.* |  | 60 | 66 | 32 ^a^ | 40 | *p=.001* |
|  |  |  |  |  |  |  |  |  |  |  |  |
| Factor 5 Worry about bf’s negative impact on father |  |  |  |  |  |  |  |  |  |  |  |
| If the mother breastfeeds, I am worried about how the father can create a close relationship with the baby. | 14 ^a^ | 11 | 4 | 8 | *ns.* |  | 13 ^a^ | 14 | 5 ^a^ | 6 | *p=.004* |
| If the mother breastfeeds, I am worried if the father feels himself to be an outsider. | 25 ^b^ | 21 | 2 | 4 | *p=.029* |  | 19 ^a^ | 21 | 8 ^a^ | 10 | *p=.012* |

^a^ one missing answer

^b^ two missing answers

^c^ three missing answers

^d^ four missing answers
